# Microneedles with Controlled Bubble Sizes and Drug Distributions for Efficient Transdermal Drug Delivery

**DOI:** 10.1038/srep38755

**Published:** 2016-12-08

**Authors:** Qi Lei Wang, Dan Dan Zhu, Xu Bo Liu, Bo Zhi Chen, Xin Dong Guo

**Affiliations:** 1 Beijing Laboratory of Biomedical Materials, College of Materials Science and Engineering, Beijing University of Chemical Technology, Beijing, 100029, P.R. China

## Abstract

Drug loaded dissolving microneedles (DMNs) fabricated with water soluble polymers have received increasing attentions as a safe and efficient transdermal drug delivery system. Usually, to reach a high drug delivery efficiency, an ideal drug distribution is gathering more drugs in the tip or the top part of DMNs. In this work, we introduce an easy and new method to introduce a bubble with controlled size into the body of DMNs. The introduction of bubbles can prevent the drug diffusion into the whole body of the MNs. The heights of the bubbles are well controlled from 75 μm to 400 μm just by changing the mass concentrations of polymer casting solution from 30 wt% to 10 wt%. The drug-loaded bubble MNs show reliable mechanical properties and successful insertion into the skins. For the MNs prepared from 15 wt% PVA solution, bubble MNs achieve over 80% of drug delivery efficiency in 20 seconds, which is only 10% for the traditional solid MNs. Additionally, the bubble microstructures in the MNs are also demonstrated to be consistent and identical regardless the extension of MN arrays. These scalable bubble MNs may be a promising carrier for the transdermal delivery of various pharmaceuticals.

Skin was considered as a potential site for systemic delivery of biopharmaceuticals with the route of transdermal delivery, which was defined as a continuous administration of pharmaceuticals across the skin^1^. Unfortunately, efficient drug delivery into the skin was restricted because of the barrier properties of stratum corneum with approximate 10 μm thickness made up dozens layers of dead cells^2^,^3^, especially for the delivery of biopharmaceuticals with high molecular weight^4^. The problem of poor drug transport via skin can be addressed by an emerging transdermal delivery system called microneedle (MN)^5^. MNs can painlessly pierce the stratum corneum and create micro channels into epidermis of the skin. Therefore, drug molecules can permeate the barrier of skin and diffuse into the subcutaneous tissues more efficiently than traditional transdermal administration^6^. Apart from the painless and mini-invasive administration^7^, the advantages of MNs for transdermal delivery include avoiding the drug degradation in digestive system compared to oral administration^6^, avoiding emotional trauma and injection risk compared to subcutaneous injection^8^, elevating the efficiency of drug delivery^4^, as well as reducing medical waste.

With the development of MEMS (micro-electro-mechanical systems)^9^, silicon^10^, metal^11^, glass^12^,^13^ and polymers^14^ have been proved to be used to fabricate MNs for the delivery of various kinds of pharmaceuticals, such as insulin^15^, growth hormone^16^, LMWH^17^, lidocaine^18^, vaccines^19^,^20^ and so on. Compared to silicon and metal MNs, dissolving microneedles (DMNs) have received increasing attention recently because of the following advantages: biocompatibility and biodegradability of the MN matrix materials, considerable drug-loading capacity, avoiding hazard tips after insertion, potentiality in mass production^21^. DMNs can encapsulate drug within a water-solvable needle matrix and release the drugs along with the dissolving of needles after insertion. Melting or solvent casting method using a MN mold was mostly used to fabricate DMNs with the melt or solution of polymers such as PVA^22^, CMC^23^,^24^, PVP^21^, PMVA^25^, silk^26^, chitosan^27^, hyaluronic acid^28^ and so on. In addition, mold free method like drawing method was also introduced to fabricate DMNs from CMC^29^, PVP^29^, PLGA^30^ and maltose^31^.

Because of the viscoelasticity of the skin and the inferior mechanical properties of DMNs, the starting insertion depth in the skin is approximately no more than one-half of the needles^32^,^33^. Therefore, it is usually difficult to achieve fully insertion of DMNs within a few minutes. In some previous communications, the drug encapsulated in DMNs usually distributes or is distributed in the whole body of the needle^34^,^35^,^36^,^37^, which accounts for the low efficiency of drug delivery into the skin and unnecessary waste. To achieve higher drug delivery into the skins, the ideal model of DMNs should encapsulate drug only in the tip of MNs and release the drug rapidly after insertion. For this purpose, several novel DMNs has been studied, such as double-layer DMNs with drug loaded in tips to increase drug utilization^38^, pedestal DMNs with supporting structures to elevate more complete insertion^29^, separable arrowhead MNs^39^,^40^, patchless DMNs^41^ and so on. Particularly, it is worth to note the bubble microneedles (BMNs) with bubble-shaped microstructures in the base of needle body. In a previous report^42^, a kind of BMNs with single-sized bubble structures was fabricated by two-step polymer coating based on a solvent casting with MN molds^42^. Technically, the bubble microstructures can prevent drug diffusion out of needle during the fabrication process and affect the drug distribution in MNs. Since the reasonable drug distribution is fundamental for effective utilization of drug in DMNs administration^43^, the control of bubble microstructures with varying dimension is worth of studying. By far, however, there are few reports investigated the control of drug distributions in MNs specifically with varying sized bubble structures.

In this work, using a new fabrication method with one-step coating process, we fabricated the BMNs with varying sized bubble structures and investigated the property of them for transdermal drug delivery. Owe to the introducing of varying-sized bubble structure, the drug distribution in BMNs were well controlled. Specifically, the size of bubbles in MNs could be well controlled by changing the concentrations of polymer solution layers during casting process, and the controlled bubble size was able to further accurately control the drug distributions in MNs. BMNs described in this work have unique advantages in improving the efficiency of drug delivery and decreasing the time of administration, which is significant for the delivery of various biopharmaceutical and rapid self-administration of MNs in future.

## Results and Discussion

### Controlled bubble size and drug distribution in MNs

**Figure 1:**
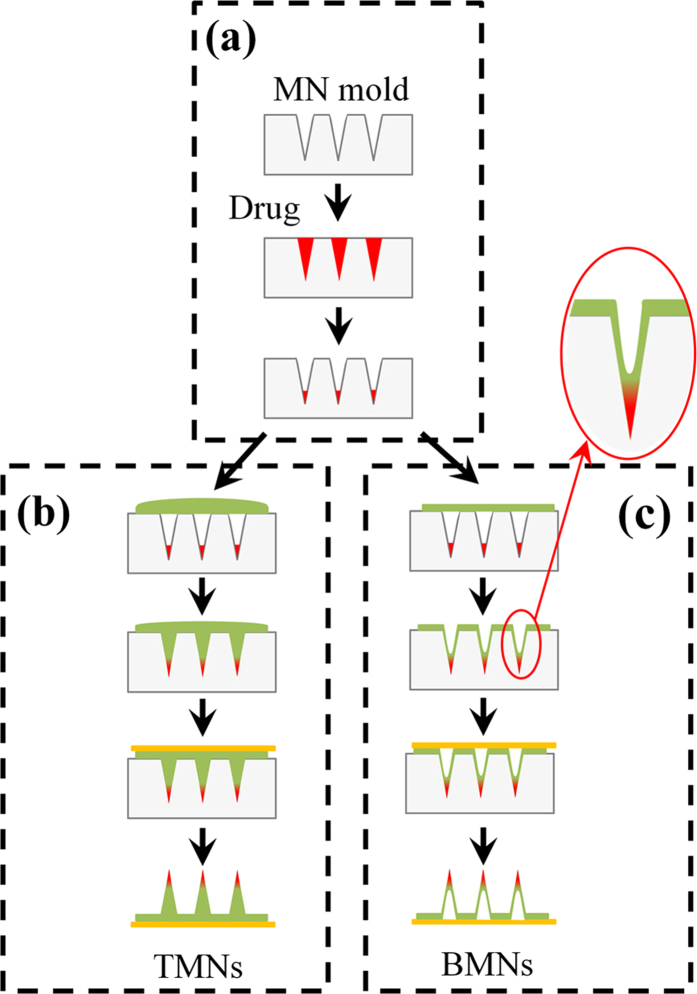
Schematic fabrication process of TMSs and BMNs. (**a**) Drug loading process. Drug solution was cast onto the mold and dried under vacuum, gathering to the tip of cavities. (**b**) Fabrication process of TMNs. PVA solution layer with traditional thickness (2 mm) was cast onto the mold filled under vacuum, and then TMNs were peeled off the mold with a base plate pasted on the backing of MNs after freeze-drying. (**c**) Fabrication process of BMNs. PVA solution layer with thin thickness (100 μm) was cast onto the mold, which was filled with thin PVA layer with sunken structures under vacuum. After freeze-drying, the dried BMNs were pasted with a base plate and peeled off the mold.

In this work, using a mold casting method, we fabricated two kinds of MNs called traditional microneedles (TMNs) and bubble microneedle (BMNs). Specially, in this work, the TMN was defined as a kind of dissolving microneedle with solid body, which was fabricated by casting sufficient matrix into the mold after drug loading process as the method shown in Fig. 1a and b. Correspondingly, BMNs was defined as the dissolving microneedle with a bubble-shaped structure in the base of MN, which was fabricated by the method shown in Fig. 1a and c. A MN mold contained conical cavities fabricated from PDMS (Polydimethylsiloxane) sheets using laser engraving technique was used^44^. Besides, Sulforhodamine B was used as the model drug, and PVA/sucrose (mass ratio 4:3) solution was used as the MN matrix. It should be noted that the PVA was the main component of MN matrix and the sucrose was used as a stabilizer for the drug. The drug loading process was show in Fig. 1a. Briefly, drug solution was put on the mold to fill the cavities under vacuum. And then the drug was dried and gathered in to the tips of cavities. After the drug loading process, to prepare TMNs with solid body of needles (Fig. 1b), 2 mm-thick layer of PVA/sucrose solution was cast onto the mold to fill the cavities under vacuum. To prepare BMMs (Fig. 1c), particularly, a thin PVA/sucrose solution layer with 100 μm thickness was cast onto the MN mold under vacuum. Due to the insufficient polymer matrix, a sunken structure was formed in the cavity under vacuum. Consequently, a void space similar to a meniscus was solidified in the body of MNs after drying. In this work, the void space at the base of MNs was defined as bubble structures. It should be noted that, compared with the previous work^42^, we employed mold with conical shaped cavities instead of pyramidal ones. It is because the former mold is favorable for the form of bubble structure and the mechanical stability of needles. In addition, the single polymer layer casting method it not only simple and efficient for fabrication but also provide the potential for the changing of bubble dimension, which would be investigated as following.

**Figure 2:**
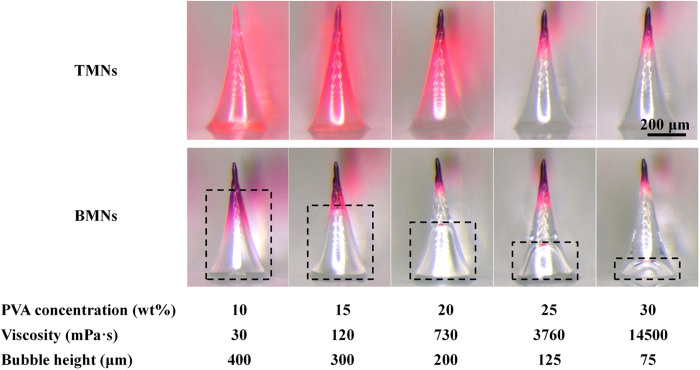
Images of TMNs and BMNs fabricated from PVA solutions with different concentrations from 10 wt% to 30 wt%. The concentrations and viscosities of PVA solutions, and the bubble heights were shown below corresponding images.

Particularly, by changing PVA concentrations (10–30 wt%) during the fabrication process of BMNs, the bubble size could be accurately controlled. The images of TMNs and BMNs with varying-size bubbles, as well as the corresponding PVA concentrations and viscosities are shown in Fig. 2. Particularly, for BMNs, when we use PVA solutions with varying concentration from 10 wt% to 30 wt% with a step of 5 wt%, the resultant heights of bubbles were 400 μm, 300 μm, 200 μm, 125 μm and 75 μm respectively. At low PVA concentrations (i.e. 10 wt% and 15 wt%), the viscosities of PVA solutions are 30 and 120 mPa•s for 10 wt% and 15 wt% PVA respectively. After PVA solution was cast onto the mold, the dried drug in the tips of the MN mold would be re-dissolved and diffused into the whole body of TMNs, due to the high drug diffusion coefficient in low PVA solutions. But for BMNs, the drug always gathered in the tip of MNs. This could be explained that the introduction of bubbles in MNs could prevent the drug diffusing from the MN tip to the MN base. When the PVA concentration increased to be 20 wt%, the viscosity of PVA solution increased to be 730 mPa•s, resulting in a decreased drug diffusion coefficient of drug in this solution. Thus, more drug distribute in the tip of TMNs as compared to those with low PVA concentrations (10 wt% and 15 wt%). Although there are still some drug diffused into the base of TMNs, the introduction of bubbles could also at least partly facilitate the gathered drug distributions in the tip of BMNs. With further increase of PVA concentrations to be 25 wt% and 30 wt%, both TMNs and BMNs had the similar drug distributions, which showed most of the drug was gathered in the MN tips. This could be explained that the high viscosities of PVA solutions, which are 3760 mPa•s and 14500 mPa•s for 25 wt% and 30 wt% PVA respectively, are the main factor that influence the drug distributions in MNs. It should be noted that higher PVA concentrations may weaken the re-dissolution of drug in the MNs fabrication process, leading to lower yield rates of MN production. Thus, it is better to fabricate the MNs with relative low PVA concentrations and gathered drug distributions in MN tips.

**Figure 3:**
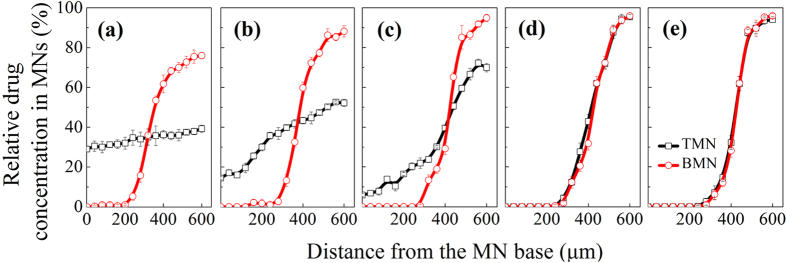
Relative drug concentrations at varying distances from the MN base. Both of TMNs and BMNs were fabricated from PVA solutions with different concentrations: (**a**) 10 wt%, (**b**) 15 wt%, (**c**) 20 wt%, (**d**) 25 wt% and (**e**) 30 wt%.

To further quantitatively estimate drug distribution profiles in MNs, the relative drug concentration at varying distances from MN bases was measured by analysing the colour depth of drug (Fig. S1). As shown in Fig. 3, when we use 10 wt% PVA solution as the MN matrix (Fig. 3a), for TMNs, the relative drug concentrations remain steady at 30–35%, indicating that the drug distributed uniformly in the whole needles. For BMNs, there was no drug loaded in the bottom of needles (0–200 μm), while the drug concentrations were increased dramatically from 0 to 75% from 200 μm to 600 μm, indicating that the drug loaded in BMNs was almost gathered in the tips rather than in the bottom. As is mentioned above, the dried drug in the tip of the mold would be re-dissolved after polymer casting process. Therefore, because of the high drug diffusion coefficient in 10 wt% PVA solutions, most of drug diffused into the whole body of TMNs. However, for BMNs, with the introduction of bubble structures, the drug would be prevented from diffusing but concentrated in the upper of the MNs. When the concentration of PVA increased to be 15 wt% and 20 wt% (Fig. 3b and c), for TMNs, with the increase of distances from MN base, the relative drug concentrations were increased slightly from 15% to 50%. It means that the drug was gradually gathered into the upper part of TMNs, due to the slightly increase of drug diffusion coefficient caused by the increase of PVA viscosity. As for BMNs, the drug concentration increased from 0 to 90% from the middle to the tip of BMNs (i.e. from 300 μm to 600 μm of the distance from the MN base). Similarly, the prevention of bubble structures should be responsible for the gathering of drug into tips of BMNs. With further increase of PVA concentrations to be 25 wt% and 30 wt%, there was little difference between TMNs and BMNs in the variation of relative drug concentration, which was higher than 90% in the tips but extremely low at the bottom of MNs. In this case, the lowest drug diffusion coefficient caused by the high viscosity of matrix was the main reason for the superior high drug distribution in the MN tips.

**Figure 4:**
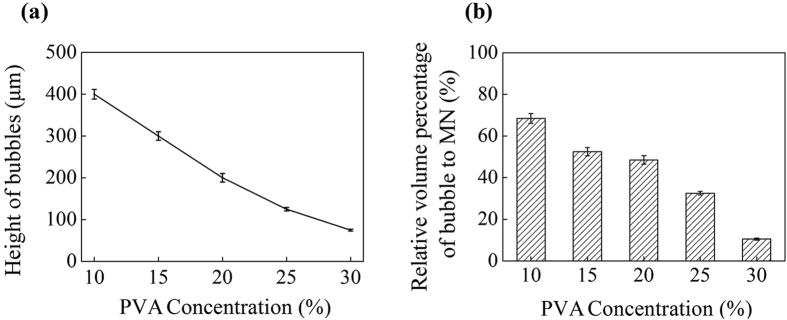
The varying bubble heights (**a**) and relative volume percentages of bubbles (**b**) in MNs corresponding varying PVA concentration from 10 wt% to 30 wt%.

As mentioned above, the introduction of bubbles in MNs could prevent the diffusion of drug from the tip to the base of MNs, which may facilitate improving drug delivery efficiency. In addition, the introduction of bubbles could also save the amount of raw materials (i.e. polymer matrix). To quantitatively estimate the percentage of raw materials which can be saved in BMNs, the volumes of bubbles and MNs were estimated as shown in Fig. S2, Supporting Information. And the relationship between bubble dimensions (the bubble height and relative volume percentage of bubble to MNs) and concentration of PVA is shown in Fig. 4. Apparently, with the increasing of PVA concentration from 10 wt% to 30 wt%, the heights of bubbles decreased from 400 μm to 75 μm approximately, and the volume percentage of bubble also decreased from 70% to 10% approximately. Due to the thin PVA solution layers with a same thickness during casting process, lower concentrations of PVA solution mean fewer amount of MN matrix. With the decrease of PVA concentration (i.e. the decrease of the amount of MN matrix), the bubbles grew bigger. Thus, due to the occupied spaces of bubbles, 10–70% materials could be saved in the production of BMNs. It is meaningful to reduce the cost of MNs, especially for expensive biomaterials.

By changing the concentrations of polymer solution, the bubble sizes and the drug distribution in BMNs could be well controlled. In addition to saving raw materials in the productions of BMNs, the bubbles may also improve the drug delivery efficiency, reducing the waste of drug and polymer matrix.

### Mechanical properties

**Figure 5:**
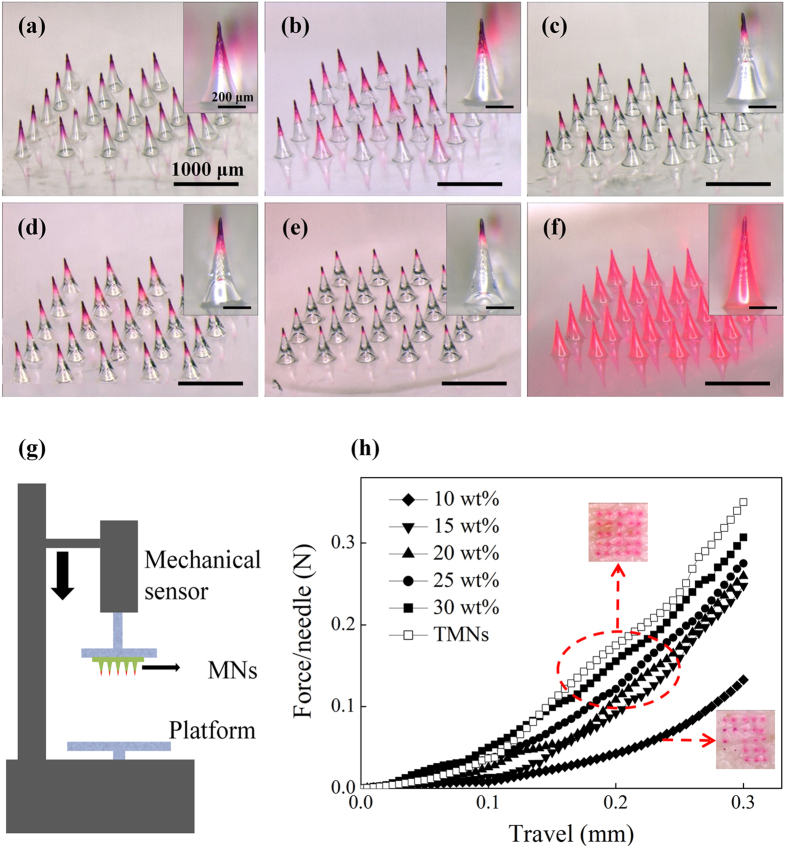
Images of BMNs fabricated from PVA solution with different mass concentration from (**a**) 10 wt%, (**b**) 15 wt%, (**c**) 20 wt%, (**d**) 25 wt% and (**e**) 30 wt% and (**f**) TMNs as a control. (**g**) Schematic of a mechanical test setup with axial moving sensor and horizontal parallel mental plate. (**h**) Mechanical behaviors of TMNs and 5 groups of BMNs fabricated from PVA solution with different concentration (10–30 wt%).

The introduction of bubbles may decrease the mechanical strength of MNs. To test if the BMNs have good mechanical strength to penetrate the skin, a micro-force test machine (Fig. 5g) was used to measure the mechanical property of BMNs. The BMNs with different bubble size (Fig. 5a–e) and TMNs (Fig. 5f) used for mechanical test are shown in Fig. 5. The images of the arrays containing 25 MNs per array for each kind of MN patch indicate that all the MNs have the uniform drug distributions and same dimensions. The mechanical behaviors of the six MN arrays are shown in Fig. 5h. The TMNs without bubble exhibited the highest mechanical strength, which was mostly caused by the solid structures of polymer matrix.

For BMNs, with the elevation of PVA concentrations (i.e. the increase of bubble size), the mechanical strength of BMNs declined gradually. Particularly, the mechanical strength of BMNs with maximum sized bubbles prepared from 10 wt% PVA was significantly lower than others. And the BMNs prepared from 30 wt% PVA had similar mechanical properties to TMNs. These results indicated that the presence of bubble could affect the mechanical properties of BMNs, the bigger of bubble was (i.e. the lower of PVA concentrations), the lower mechanical strength of MNs we obtained. In further skin insertion tests, the BMNs prepared from 15 wt% PVA solution were demonstrated to be inserted into the skin completely, as shown in the image of porcine skin in Fig. 5h. However, the BMNs prepared from 10 wt% PVA showed inferior insertion capacities, which were indicated by 75% effective insertions in the skin (Fig. 5h). This difference could be explained like that, for the BMNs prepared from 15–30 wt% PVA, attributing to the arch structures of the top of bubbles, the axial compression was distributed uniformly onto the “walls” of BMNs during the mechanical test or skin insertion test. However, when the PVA concentration decreased to 10 wt%, the bubble dimension was increased and the thickness of the walls around the bubbles become extremely thin, which accounted for the apparent reduction of the mechanical strength and the failure of insertion on the model skin. Above all, with the growth of bubbles, the thickness of the wall around bubbles would be reduced, which might not provide a strong support to BMNs when applied in the skin. Thus, it is easy to see the significance of the control of bubble size in MNs. Although bubble structures could prevent the diffusing of drug to MN base, it does not mean the bigger bubbles are beneficial to the properties of BMNs. To insure reasonable insertion capabilities of BMNs, the minimum concentration of PVA solution is 15 wt%.

### *In vitro* insertion test to porcine skin

**Figure 6:**
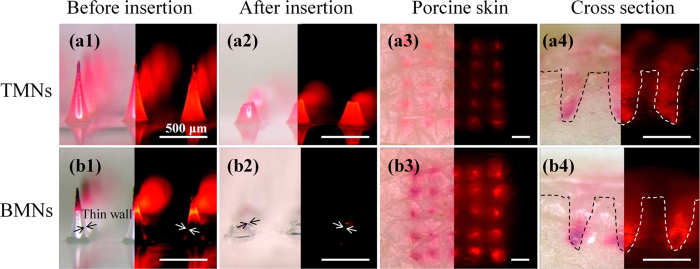
Images BMNs (a1, a2) and TMNs (b1, b2) prepared from 15 wt% PVA solution before (a1, b1) and after (a2, b2) insertion into the skin for 30 s and porcine skin after application of TMNs (a3) and BMNs (b3). All the left half of images were shown under visible light, and the right half of the images were shown under fluorescence.

To evaluate the insertion capability of TMNs and BMNs *in vitro*, porcine skin was used to test the insertions of drug-loaded TMNs and BMNs prepared from 15 wt% PVA solutions. In this test, the MNs were inserted into the skin and removed after 30 s using an in-house made applicator. As shown in Fig. 6, the TMNs and BMNs before and after insertion, as well as the skins after 30 s insertion were observed under visible light (left half of images) and fluorescence (right half of images). For TMNs, the drug distributed in the whole body of solid needle (Fig. 6a1), and a mass of drug remained in the residual base (about 250 μm in height) after insertion (Fig. 6a2). For BMNs, however, with the bubble-shaped structures in each needle, the drug was mostly encapsulated in the MN tips, and the “thin wall” structures were generated in the outer layer of BMNs (Fig. 6b1). After application in the porcine skin, there was little drug left in the residual base (about 100 μm in height), which was much shorter than that of TMNs (Fig. 6b2). In addition, as shown in the images of porcine skin (Fig. 6a3 and b3), the red holes indicated the successful pierce of the skin and drug release after the application of TMNs and BMNs. But the insertion effect of BMNs is apparently better than the former, indicating by the deeper color intensity in the holes generated by MNs. Moreover, from the cross section view of skins (Fig. 6a4 and b4), it is found that the drug delivered by TMNs was distributed all over the needle holes. While for the BMNs, most drugs were delivered into the bottom of holes. The result described above demonstrated that, both of TMNs and BMNs prepared from 15 wt% PVA had good insertion capabilities, while the latter showed more effective drug delivery into the skin. This difference could be explained by two main reasons. On one hand, when the MNs were inserted into the skin, the tips or the upper part of MNs were dissolved firstly. Compared with the wide drug distribution in the whole TMNs, the concentrated drug in the tips of BMNs enabled more all the drug penetrated into the subcutaneous tissues after insertion. On the other hand, the “thin wall” structures caused by bubbles could be dissolved rapidly in the skin, which accelerated the separation of tips from bases. Therefore, fewer residual MN bases remained and more drug was delivered into the skin with the same insertion time. These results indicated that the BMNs would be potential in elevating the utilization of drug.

### Efficiency of drug delivery

**Figure 7:**
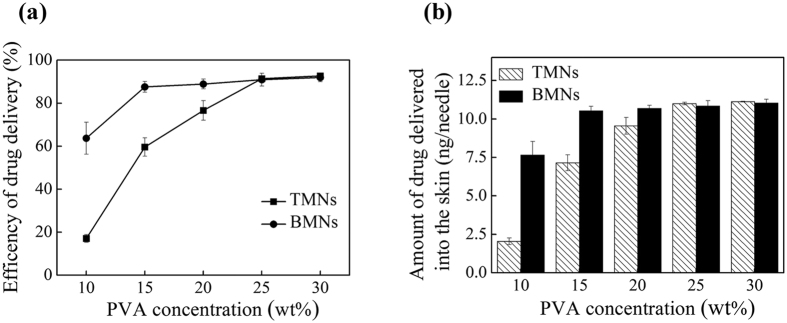
Efficiency of drug delivery (**a**) and amount of drug delivered into the skin (**b**) after insertion in 30 s with BMNs and TMNs fabricated from PVA solution with mass concentration from 10 wt% to 30 wt%.

As described above, the mechanical properties and insertion capabilities of TMNs and BMNs were investigated. And we found the BMNs showed superior visible efficient drug delivery into the skin. To further evaluate the efficiency of drug delivery quantitatively, the amount of drug delivered into the skin was measured after 30 s insertion. It should be noted that we used 1 mg/mL Sulforhodamine B solution and in the fabrication, and the TMNs and BMNs were prepared from PVA solution with varying concentrations of 10 wt%, 15 wt%, 20 wt%, 25 wt% and 30 wt%. The drug loading is 12 ng/needle. The efficiency of drug delivery (i.e. the ratio of amount of drug delivered into the skin to the total drug loading in the MNs) and amount of drug delivered into the skin was obtained and shown in Fig. 7a and b respectively. When using PVA solution with low concentrations (i.e. 10 wt%, 15 wt% and 20 wt%), for BMNs, the efficiencies of drug delivery were 63%, 85% and 88% respectively, and the amounts of drug delivered into the skin were about 7.6 ng, 10.5 ng and 10.7 ng (per needle) respectively. For TMNs, the corresponding efficiencies of drug delivery of were 15%, 59% and 76% respectively, and the amounts of drug delivered into the skin were about 2.1 ng, 7.1 ng and 9.5 ng (per needle) respectively, which are obvious lower than those in BMNs. For the TMNs, the drug molecules always diffuse into the whole body of the MNs during the polymer casting process, due to the low PVA concentrations. For BMNs, the presence of bubbles could prevent the diffusing of drug from the MN tips to the MN bases even at low PVA concentrations, i.e. facilitated the gathering of drug into the MN tips, which would be dissolved under the skin firstly. For this reason, more drug could be delivered into the skin when we use BMNs. Nevertheless, there was little difference between TMNs and BMNs in the efficiency or the amount of drug delivery when we use 25 wt% and 30 wt% PVA solution. As is described previously, because of high PVA concentrations, the drugs loaded in two kinds of MNs were well gathered into tips in a same manner regardless the presence of bubbles. Therefore, both of TMNs and BMNs showed considerable efficiency of drug delivery when using higher concentration PVA.

### Kinetic dissolution of MNs in the skin *in vivo*

**Figure 8:**
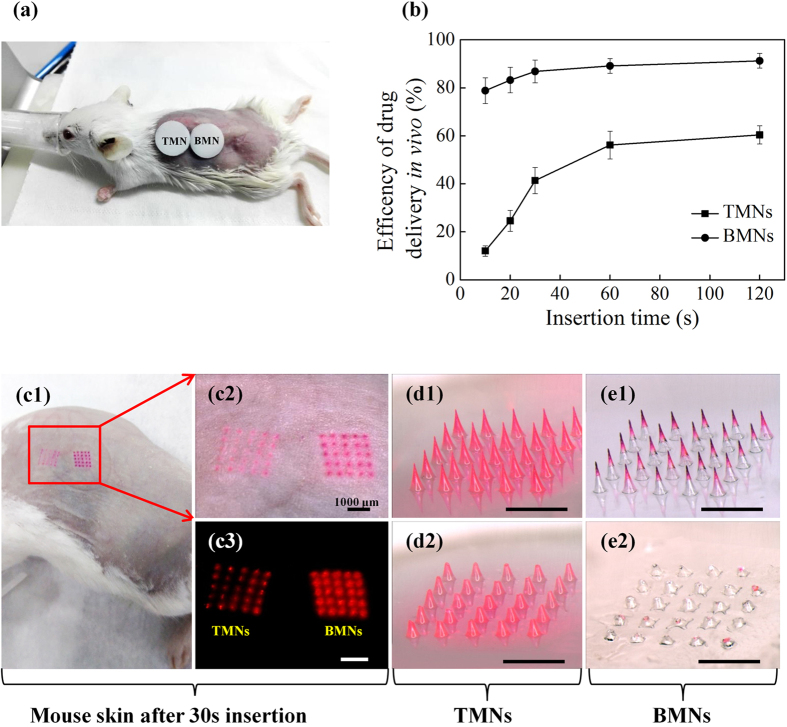
(**a**) The illustration of the dehairing moues treated with TMNs and BMNs patches prepared from 15 wt% PVA solution on the back skin. (**b**) The efficiency of drug delivery *in vivo* within varying insertion time (i.e. 10 s, 20 s, 30 s, 60 s and 120 s). The illustration of the mouse skin after 30 s insertion (c1, c2 and c3), and the corresponding images of TMNs before (b1) and after insertion (b2), as well as the BMNs before (c1) and after insertion (c2).

Considering the practical applications of MNs for drug delivery in the future, it is meaningful to shorten the administration time of MN to obtain more compliance and reliance for patients. So we except the BMNs would enable a rapid dissolution of MNs to achieve efficient drug delivery. To test the exception, we evaluated the kinetic dissolution of TMNs and BMNs under mice skin *in vivo,* i.e. the efficiency of drug delivery within a specific insertion time (i.e. 10 s, 20 s, 30 s, 60 s and 120 s). In this study, the TMNs and the BMNs, both prepared from 15 wt% PVA solutions, were inserted into a mouse skin (Fig. 8a). After the specific insertion time, the efficiency of drug delivery was measured and shown in Fig. 8b. Specifically, the treated skins after 30 s insertion were illustrated in Fig. 8c1–c3. In addition, the TMNs before and after 30 s insertion were shown in Fig. 8d1 and d2, as well as the BMNs before and after 30 s insertion were shown in Fig. 8e1 and e2.

From Fig. 8b, it can be observed that the drug delivery efficiencies of TMNs were 12%, 24%, 41% within 10 s, 20 s and 30 s insertion time respectively, and no more than 60% even within 2 min. However, for BMNs, the efficiencies were 79%, 83%, 86% within 10 s, 20 s and 30 s insertion time respectively, and higher than 90% within 1 min and 2 min insertion. Therefore, the BMNs showed shorter administration time and higher efficient drug delivery as compared to the TMNs. As shown in the images of the mouse skin under visible light (Fig. 8c2) and fluorescence (Fig. 8c3), it is apparent that the BMNs caused clearer red holes after 30 s insertion compared to TMNs. To explain this, the MNs before (Fig. 8d1 and e1) and after (Fig. 8d2 and e2) insertion were collected and observed under a microscope. For TMNs before insertion (Fig. 8d1), the drug was widely distributed in the MNs even diffused into the backing layer of the MN patch. And after 30 s insertion (Fig. 8d2), there was some drug residues still left in the MN base and the backing. Therefore, only a part of drug was delivered from TMNs into the skin, which account for the low drug delivery efficiencies *in vivo*. For BMNs, the residual drug in the backing after 30 s insertion was barely visible (Fig. 8e2). This could be explained that the drug was concentrated on the upper or the tips of BMNs (Fig. 8e1), which is largely ascribed to the barrier function of bubble structures. When penetrated into the skin, the thin layer at the bubble location would be quickly dissolved, leading to the separation of drug loaded MN tips and the MN bases bellow the bubbles. Thus, BMNs showed a more efficient drug delivery as compared to TMNs within an equal insertion time.

### Fabrication of scalable BMN arrays

**Figure 9:**
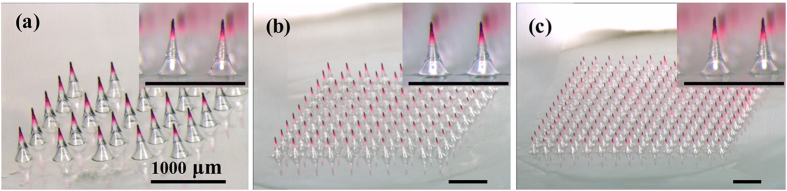
Images of (**a**) 5 × 5, (**b**) 10 × 10 and (**c**) 15 × 15 BMN arrays and their details with enlarged scale.

Considering the future applications of BMNs, it is also significant to enlarge the production according to various practical requirements. Therefore, to further evaluate the feasibility of the scalable fabrication, we prepared 5 × 5, 10 × 10 and 15 × 15 BMN arrays encapsulating Sulforhodamine B using 20 wt% PVA solutions as the matrix. As presented in Fig. 9, the bubble microstructures of BMNs in the three groups are consistent and identical regardless the extension of MN arrays. In other words, the enlarged production of BMN arrays would not influence the favorable geometries of MNs and bubble structures. Consequently, the fabrication method of BMNs described in this paper is significant for the industrialized production in future. Moreover, the scalable fabrication of BMN patches would be also meaningful to meet the practical drug doses requirements for various pharmaceuticals.

## Materials and Method

### Materials

PVA (polyvinyl alcohol, 75% hydrolyzed, MW approximately 2000) was purchased from Acros Organics (New Jersey, USA). Sucrose (MW approximately 324.3) was purchased from Tokyo Chemical Industry Company (Tokyo, Japan). Sulforhodamine B was purchased from Lakeshore Biomaterials (Birmingham, AL). Porcine cadaver skins were purchased from a local slaughterhouse immediately after death.

### Fabrication of TMNs and BMNs

In this work, solution casting method base on MN molds was used to prepare the MNs. PVA was mixed with sucrose with mass ratio 4:3, and then dissolved in a specific volume of DI water to prepared the MN matrix with varying PVA mass concentrations (i.e. 10 wt%, 15 wt%, 20 wt%, 25 wt% and 30 wt%). Sulforhodamine B used as a model drug was dissolved in DI water to get the 1 mg/mL drug solution. The MN molds were prepared on the surface of PDMS sheet using laser micromachining technique. In this study, we used the 5 × 5 MN arrays with height of 600 μm and base diameter of 300 μm. The fabrication of MNs was started with the drug loading process (Fig. 1a). Firstly, 1 mg/mL model drug was put on the surface of PDMS mold to fill the cavities under vacuum. After removal of redundancy, the solution in the cavities was dried under vacuum and concentrated to the tips of MN molds. To prepare drug-loaded TMNs with no bubbles, PVA/sucrose solution with an approximate thickness of 2 mm was cast on the surface of mold. And then the cavities of mold were filled under vacuum. After that, TMNs was freeze-dried under vacuum and peeled off with an adhesive plate pasted on the surface (Fig. 1b). In contrast with TMNs, BMNs possess bubble-shaped microstructures in the bottom of needles instead of solid structures wholly. To obtained bubble structures, a thinner PVA solution layer with 100 μm of thickness was cast on the surface of PDMS mold. Therefore, the cavities in molds were filled by thin PVA layers with sunken structures under vacuum. And then, after freeze-drying process, the resultant BMNs was peeled off directly with an adhesive plate on the base (Fig. 1c). It should be particularly mentioned that, five groups of PVA solution with different concentrations (10 wt%, 15 wt%, 20 wt%, 25 wt% and 30 wt%) were used to control the bubble size accurately during the fabrication process. Both of TMNs and BMNs were observed using an optical/fluorescence microscope (Olympus SZX7, Japan).

### Drug distribution in MNs

Using an optical microscope, we could observe different drug distributions within TMNs and BMNs prepared from different MN matrix. In addition, a color analyzing software (Color Pix, Color Schemer 2003) was used to estimate the relative drug concentration by color depth of drug at varying location in MNs. Specifically, as shown in Fig. S1, supporting information, we transformed the microphotographs of drug-loaded MNs into grayscale, and then we obtained black color values at each part using the color analyzing software. The color values of black displayed in “Color Pix” range from 0 to 100. In this work, we defined the color value at the part of MNs without drug as 0, and higher color value means higher drug concentration. Resultant relative drug concentration at varying distances from MN base was obtained using the ratio of color values to max value (100). Five kinds of TMNs or BMNs prepared from different PVA solutions (10 wt%, 15 wt%, 20 wt%, 25 wt% and 30 wt%) was investigated with this method.

### Mechanical property test

To investigate the mechanical properties of MNs with different sized bubbles, we used a micro-force test machine with an axial moving mechanical sensor (Series 5 Digital Force Gauge, MARK-10 Corporation, Copiague, USA) and a metal platform. The MN arrays were attached on the bottom of mechanical sensor, which moved axially at a constant rate of 0.5 mm/min. The forces were measured when the uppermost tip of MN touched the platform. The travels of MNs (i.e. the travels of mechanical sensor) and forces were recorded in the computer.

### Skin penetration tests

Porcine skin was used as a model skin for skin penetration tests of TMNs and BMNs to evaluate the capability of insertion and drug release *in vitro*. Briefly, porcine cadaver was washed by 75% ethanol after shaving, and then MN arrays was pressed onto the porcine skin in a vertical direction using a force of approximate 10 N. After specific insertion time (i.e.10 s, 20 s, 30 s etc.), the MN arrays were removed from the surface of the skin. Then the MNs and skins were observed under optical/fluorescence microscope with visible light and fluorescence.

### The efficiency of drug delivery of BMNs and TMNs

To evaluate the efficiency of drug delivery of TMNs and BMNs into the skin, a fluorescence microplate reader (Thermo Fisher Science OY, Vantaa, Finland) was used to measure the amount of drug delivered into the skin. Briefly, TMNs and BMNs were inserted into porcine skin with the same force and removed from skin after a specific time (i.e.10 s, 20 s, 30 s etc.). And then, the drugs remained in MN bases and the outer surface of the skin were collected and re-dissolved in a specific volume of DI water and measured by a microplate reader. In this work, we defined the percentage of drug delivery as the ratio of amount of drug delivered into the skin to the total drug loading in the MNs.

### *In vivo* transdermal delivery to mouse skin

To validate the feasibility of the MNs for transdermal delivery *in vivo* and evaluated the kinetic dissolution of MNs under skin, female BALB/C mice, 6–8 weeks old, 16 ± 0.7 g) were used as administration model. And 5 × 5 arrays of drug loaded TMNs and BMNs with 600 μm height were inserted into the depilated mice skin using an in-house made applicator and removed after specific time (i.e.10 s, 20 s, 30 s etc.). And then, an optical/fluorescence microscope was used to visualize the mice skin applied by TMNs and BMNs. The methods were carried out in accordance with guidelines and regulations the internal experiment ethic committee of Beijing University of Chemical Technology. All animal experiments were approved by the ethic committee of Beijing University of Chemical Technology and the National Institutes of Health.

## Conclusions

A novel fabrication method of bubble microneedles with controlled internal bubble structures and drug distributions is proposed in this work. With the introduction of bubble structures, the BMNs showed a special good performance in controlling the drug distributions and increasing the drug delivery efficiencies. When inserted into the skin, the drug encapsulated in the tips of BMNs was dissolved and released rapidly due to the quick dissolution of the thin polymer layer at the bubble location. Compared to TMNs, the BMNs showed higher efficiency of drug delivery both *in vitro* and *in vivo*, as well as a shorter administration time. We expect that the BMNs with controlled drug distributions could provide a progressive route in the encapsulation and delivery of various therapeutic drugs like vaccines and proteins, as well as promote the reliability of MNs for transdermal drug delivery in future.

## Additional Information

**How to cite this article**: Wang, Q. L. *et al*. Microneedles with Controlled Bubble Sizes and Drug Distributions for Efficient Transdermal Drug Delivery. *Sci. Rep.* **6**, 38755; doi: 10.1038/srep38755 (2016).

**Publisher's note:** Springer Nature remains neutral with regard to jurisdictional claims in published maps and institutional affiliations.

## Supplementary Material

Supplementary Information
