# Supplementary material for: Microneedles with Controlled Bubble Sizes and Drug Distributions for Efficient Transdermal Drug Delivery
Source: Sci Rep. 2016 Dec 8;6:38755. doi: 10.1038/srep38755 (PMC5144082; doi:10.1038/srep38755)
Supplement: Supplementary Information [file srep38755-s1.doc]

Supplementary information for

**Microneedles with Controlled Bubble Sizes and Drug Distributions for Efficient Transdermal Drug Delivery**

*Qi Lei Wang^1^, Dan Dan Zhu^1^, Xu Bo Liu, Bo Zhi Chen, Xin Dong Guo**

Beijing Laboratory of Biomedical Materials, College of Materials Science and Engineering, Beijing University of Chemical Technology, Beijing, 100029, P.R. China.

^1^They contributed to the work equally.

Corresponding author’s E-mail: [xdguo@buct.edu.cn](mailto:xdguo@buct.edu.cn)

**Figure S1.** Illustrations of the estimation of the drug distributions using color depth. The instances of the measurement of black color values at varying distances from MN base on the images of (a) TMNs and (b) BMNs.

**Figure S2.** Simulated images of BMNs with varying heights and volumes calculated by Unigraphics NX.
